# Comparison of Three Methods Used in the Diagnosis of Extraesophageal Reflux in Children with Chronic Otitis Media with Effusion

**DOI:** 10.1155/2015/547959

**Published:** 2015-05-06

**Authors:** Martin Formánek, Pavel Komínek, Petr Matoušek, Radoslava Tomanova, Ondřej Urban, Karol Zeleník

**Affiliations:** ^1^Department of Otorhinolaryngology, University Hospital Ostrava, 17 Listopadu 1790, 708 52 Ostrava, Czech Republic; ^2^Faculty of Medicine, University of Ostrava, Syllabova 19, 703 00 Ostrava, Czech Republic; ^3^Department of Pathology, University Hospital Ostrava, 17 Listopadu 1790, 708 52 Ostrava, Czech Republic; ^4^Gastroenterology Department, Vitkovicka Hospital, Zalužanského 1192/15, 703 84 Ostrava, Czech Republic

## Abstract

*Objectives*. Detection of extraesophageal reflux (EER) in children with chronic otitis media with effusion (OME) using three different diagnostic methods.* Methods*. Children between 1 and 7 years with OME who underwent adenoidectomy and myringotomy with insertion of a ventilation tube were included in this prospective study. EER was detected using three methods: oropharyngeal pH was monitored for 24 hours using the Restech system; detection of pepsin in middle ear fluid obtained during myringotomy was done using Peptest, and detection of pepsin in an adenoid specimen was done immunohistochemically.* Results*. Altogether 21 children were included in the study. Pathological oropharyngeal pH was confirmed in 13/21 (61.9%) children. Pepsin in the middle ear fluid was present in 5/21 (23.8%) children; these 5 patients were diagnosed with the most severe EER established through monitoring of oropharyngeal pH. No specimen of adenoids tested was positive for pepsin upon immunohistochemical examination.* Conclusions*. Diagnosis of EER in patients with OME using Restech is sensitive but less specific when compared to the detection of pepsin in middle ear fluid using Peptest. Pepsin in the middle ear was consistently present in patients with RYAN score above 200, and these patients in particular could potentially profit from antireflux therapy.

## 1. Introduction

Acute otitis media (AOM) and chronic otitis media with effusion (OME) are among the most frequent causes for visits to the doctor in children 1–3 years old. Despite of the fact that there was an overall downward trend in the United States during the pneumococcal conjugated vaccine era, AOM and OME remain major health and socioeconomic issue [1]. It is estimated that up to 60% of children have experienced at least one episode of AOM by age 7 [2, 3]. There are several well-known conditions that cause or facilitate the development of middle ear infection. The most important are upper respiratory infections, allergies, and enlarged adenoids [4]. Despite adequate treatment of these conditions, AOM and OME remain common issues [5, 6]. In consequence, there is an effort to identify other possible risk factors and thereby reduce the number of ear infections and their consequences.

Extraesophageal reflux (EER) is considered one among several possible risk factors of AOM and OME [5–9]. Until recently, more accurate exploration of the relationship between ear infection and EER has been very complicated due to limitations in diagnostic methods. However, in recent years superior pharyngeal pH monitoring devices and new techniques which can measure pepsin in tissues and fluids have been developed and EER can be diagnosed quite precisely [10, 11]. The problem is that it is not yet known how to select patients with OME who would respond to antireflux therapy. The reason for this is that a diagnosis of pathological EER based on the given thresholds does not mean that the patient will respond to antireflux therapy [12]. And because of likely side effects, it is not possible to put all patients with OME on proton pump inhibitors.

The aim of the study was detection of EER in children with OME using three different diagnostic methods (oropharyngeal pH monitoring, detection of pepsin in the middle ear fluid, and immunohistochemical detection of pepsin in a biopic specimen of adenoids) and selection of the group of patients with the most severe EER who could potentially benefit from antireflux therapy (diet, behaviour, and proton pump inhibitors).

## 2. Materials and Methods

The prospective study was approved by the Ethics Committee of the University Hospital and was performed in accordance with the Declaration of Helsinki, good clinical practice, and applicable regulatory requirements. Written informed consent was obtained from both parents before initiating any procedure.

Children aged between 1 and 7 years diagnosed with bilateral or unilateral OME who underwent adenoidectomy and myringotomy with insertion of a ventilation tube were included in the prospective study from June 2012 to March 2014. OME was defined as effusion in the middle ear behind an intact eardrum for longer than 3 months. Diagnosis was made on the basis of otomicroscopic findings, pneumatic otoscopy, type B tympanometry, and audiometry (in cooperative older children). Children with no fluid in the middle ear during myringotomy were rediagnosed as having tympanosclerosis and were excluded from the study. Children with craniofacial abnormalities (Down syndrome, Treacher Collins syndrome, clefts, etc.) were excluded from the study as well. Demographic data (including tobacco exposure) and symptoms of EER disease were provided by parents, who were also specifically questioned regarding the presence of hoarseness, recurrent lower respiratory infection (e.g., bronchitis and pneumonia), and bronchial asthma in their child.

24-hour monitoring of oropharyngeal pH using the Restech system (Respiratory Technology Corporation, San Diego, CA, USA) was performed before surgery. Parents were instructed to record the time their child spent eating and drinking and in a horizontal position directly to the device and manually to the diary. If there was any discrepancy, periods logged in the device were modified according to the diary. A standardized RYAN composite score was calculated automatically using the software supplied. Patients with pathological RYAN composite scores in the vertical (higher than 9.4) and/or horizontal (higher than 6.8) position were classified as having pathological EER. Severe EER was diagnosed when the RYAN composite score in the vertical or horizontal position was higher than 200.

Myringotomy under magnification was performed in the anterior inferior part of the tympanic membrane. The type of middle ear effusion (i.e., fluid or mucous) was noted. Middle ear fluid was collected with a Tympanocentesis Collector 1419020 (Medtronic, Minneapolis, MN, USA), and a ventilation tube was inserted in the tympanic membrane. In cases of bilateral OME, bilateral ventilation tube insertion was performed simultaneously and the effusion was collected and analyzed separately. Analyses were performed on the day of surgery. First, 0.1 mL of 10% citric acid was added. Afterwards, the specimen was centrifuged at 4,000 rpm for 5 min. If a clear supernatant layer was not visible, the sample was centrifuged again. An 80 *μ*L sample was drawn from the clear supernatant layer, added to a screw top microtube containing 240 *μ*L of migration buffer, and mixed with a vortexer for 10 s. Afterwards, the specimen was assayed with Peptest (RD Biomed Limited, Hull, UK), which contains monoclonal antibodies targeted to pepsin. The results were collected after 15 min. Peptest results are specified as positive (two lines), negative (one line), or invalid (no line).

Then, adenoidectomy using a cold instrument was performed. A specimen of adenoids (5 × 5 × 5 mm) from the area close to the torus tubarius was fixed in formaldehyde and immunohistochemically analysed at the Department of Pathology. Antibody P3635Rb-h (Uscn Life, USA, concentration 1 : 100) was used as the primary antibody. Antibody N-Histofine Simple Stain MAX PO (Nichirei Biosciences Inc., USA) was used as the secondary antibody. Statistical analysis was done using MS Excel. There was no missing data.

## 3. Results

In total, 24 children were included in the study. Three children with no middle ear fluid during myringotomy were rediagnosed as having tympanosclerosis and were excluded from the study. Thus 21 children, 11 boys (52.4%) and 10 girls (47.6%), with an average age of 4.2 years, were analysed. 2/21 (9.5%) children were hoarse and were diagnosed with vocal cord nodules, 3/21 (14.3%) suffered from recurrent pneumonias (3 or more pneumonias during the previous two years), and 5/21 (23.8%) children suffered from bronchial asthma. None of the children took medications for gastroesophageal reflux disease.

Pathological EER was diagnosed by oropharyngeal pH monitoring (Restech) in 13/21 (61.9%) children. The average RYAN composite score of patients diagnosed with EER was 106.05 in the vertical position and 6.69 in the horizontal position. In 5/21 (23.8) children, the RYAN composite score in the vertical position was higher than 200 (severe EER).

Bilateral myringotomy was performed in 12/21 (57.1%) children and unilateral myringotomy in 9/21 (42.9%) children. Altogether, 33 middle ear fluid specimens were examined. Pepsin in the middle ear was detected in 5/21 (23.8%) children. In three children with bilateral OME, pepsin was detected in the middle ear fluid in both ears. Pepsin was detected in the middle ear fluid in two patients with unilateral OME as well. Thus pepsin was detected in 8/33 (24.2%) middle ear specimens. No invalid result was noted. Serous samples were positive to pepsin in 5/17 (29.4%) cases, while mucous samples were positive in 3/16 (18.8%) cases. Pepsin in the middle ear fluid was present only in 5 children with severe EER (RYAN composite score higher than 200), as established by monitoring the oropharyngeal pH. In the remaining 8 children with less serious EER ascertained by means of oropharyngeal pH monitoring, pepsin in the middle ear fluid was not diagnosed. Pepsin in the middle ear was detected in 2/5 children (40.0%) with bronchial asthma.

Immunohistochemical detection of pepsin in biopic specimens of adenoids was negative in 21/21 (100%) samples. Antibodies used for control in the main cells of the gastric mucosa were strongly positive.

## 4. Discussion

It is supposed that EER is an etiological factor or cofactor in many lower and upper respiratory diseases, such as laryngitis, cough, globus pharyngeus, bronchial asthma, papillomatosis, and rhinosinusitis, and in middle ear inflammations, as well [13, 14]. Many studies investigated that contact between the refluxed content and mucous of the nasopharynx, Eustachian tube, or middle ear causes local inflammation and oedema; thus it facilitates the development of middle ear inflammation [5–9, 15]. This is why EER is nowadays included among other well-known predisposing factors for developing middle ear inflammation [5–9, 15].

Diagnosis of EER in patients with OME is not easy. Many reflux questionnaires have been developed in the recent past, even for infants and small children. They summarize complaints potentially caused by reflux (frequent awakening at night, regurgitation of food, hoarseness, cough, lower respiratory infections, etc.) [16, 17]. However, evaluation of reflux, and particularly EER in children using questionnaires, seems to be inadequate and inaccurate, because symptoms are very common and too heterogeneous [16]. Another problem is that the questionnaire is filled in by parents, who could interpret symptoms incorrectly. For children older than 12 years, the Reflux Symptom Index can be used to evaluate patient problems [16].

Many novel methods have become available recently for making the diagnosis of pathological EER and quantifying it. Diagnosis of EER by 24-hour esophageal pH-metry or impedance is relatively invasive and not always well tolerated, especially by children. Therefore it is advantageous to use new, less invasive diagnostic methods, such as 24-hour monitoring of oropharyngeal pH by the Restech system, detection of pepsin in middle ear fluid using Peptest, and immunohistochemical detection of pepsin in tissues.

Currently, one of the widely used methods for measuring EER is 24-hour monitoring of pH in the esophagus. It has been shown that there is a 10 times higher risk of development of recurrent AOM or OME in children in whom EER is detected by means of double-probe esophageal pH monitoring [9]. However, double probe esophageal pH monitoring is not very well tolerated by children, especially children aged two to seven years. This is one of the reasons why oropharyngeal pH monitoring, which is less invasive and much better tolerated by children, was developed and implemented in clinical practice [11]. However, there are some disadvantages of this method as well. In particular, the absence of a distal sensor, which means that it is necessary to rely on data about meal periods and the position of the patient as entered by the parent. Nevertheless, the majority of studies comparing esophageal and oropharyngeal pH-metry (simultaneous monitoring in one patient) have established good reciprocal correlation between these two methods [10, 18].

There is no pepsin in the middle ear in normal physiologic conditions [5]. The presence of pepsin in the middle ear is therefore considered indirect confirmation of previous episodes of reflux into the middle ear [5, 6]. In the study by O'Reilly et al. pepsin in middle ear effusions in patients with recurrent AOM or OME was detected in 20.2% of cases, in comparison with the control group of patients who underwent cochlear implantation (only 1.5% cases) [7]. Other studies that examined pepsin in the middle ear secretions of children with OME refer to the presence of pepsin in 1/3 cases [8]. This suggests that EER is likely one of the etiological factors behind OME in as many as 1/3 children. Similar results were obtained in our study, as pepsin was detected by Peptest in 5/21 (23.8%) children, more frequently in serous samples (29.4%) than in mucous samples (18.8%). Previous studies use accurate but time consuming and expensive methods of detecting pepsin, which are too complicated to be used on a daily basis. Peptest, on the other hand, seems to be suitable for frequent daily use as an easy, cheap, and quick diagnostic method.

It is possible to detect pepsin in tissues using immunohistochemical analysis as well [15]. In the study by Jiang et al., immunohistochemical detection of pepsin in interarytenoid biopsy specimens in patients with pathological EER (detected by esophageal impedance) was performed. In their study, pepsin was evidenced both in patients with acid (6 of 7 patients) and with weak acid reflux (6 of 8) [15]. Pepsin was evidenced in 3/21 patients in the control group who had negative results for esophageal impedance. This can be explained by the higher sensitivity of an immunohistochemical examination due to the protracted collection of pepsin in tissues, compared to pH monitoring that lasts only 24 hours. It is possible to detect pepsin in tissues even though there may have been no reflux over several days [15]. In theory, the diagnosis of pepsin in adenoids could be another way to diagnose EER in children with OME so as to get a wider view of the severity of reflux in the nasopharynx. Interestingly, in our study, all 21 specimens of adenoids were found to be pepsin negative using immunohistochemical detection. The authors cannot explain this fact but only speculate that the amount of pepsin in the nasopharynx was too low to be detected (in comparison with the interarytenoid region). Our results are consistent with the results of Harris et al., where pepsin was not detected in specimens of adenoids, and the authors conclude that this method is not suitable for the diagnosis of EER in the nasopharynx [19].

All in all, using 24-hour monitoring of oropharyngeal pH (Restech) and detection of pepsin in the middle ear fluid (Peptest), diagnosis of EER in patients with OME and its quantifying can be accomplished quite precisely nowadays. But there is still one big question remaining to be answered: which patients would respond to antireflux therapy? The problem is that AOM/OME, as well as EER, are very common diseases, and diagnosis of pathological EER according to the given thresholds does not guarantee that the patient will respond to antireflux therapy. Last systemic review of Miura et al. concludes that the prevalence of gastroesophageal reflux disease in children with chronic otitis media with effusion/recurrent acute otitis media may be higher than the overall prevalence for children. However, presence of pepsin/pepsinogen in the middle ear could be related to physiologic reflux. A cause-effect relationship between pepsin/pepsinogen in the middle ear and otitis media is unclear and therefore antireflux therapy for otitis media cannot be endorsed based on existing research [20]. And because it is not possible to put all patients with OME on proton pump inhibitors, particularly because of possible side effects, it is very important to quantify EER. It has been proved that the stricter the criteria for the diagnosis of EER, the more the patients that would respond to antireflux therapy [12]. The results of our study demonstrated that pepsin in the middle ear fluid was present in five children with the most severe EER (RYAN score above 200) established by monitoring of oropharyngeal pH. On the contrary, eight children with mild pathological EER had no pepsin in their middle ear fluid. In order to select patients with severe EER, who would potentially benefit from antireflux therapy, this information seems to be very important. It can be assumed that patients with a RYAN composite score above 200 and patients with a positive Peptest would be the best candidates for antireflux therapy. Whatever the case, it is very important to pursue research in this area with better designed controlled studies with more patients involved.

## 5. Conclusions

EER can cause inflammatory changes in the Eustachian tube and middle ear, with consequential development of middle ear inflammation. On the basis of previous studies, as well as ours, we may conclude that EER is likely coresponsible for as many as 1/3 of OME. 24-hour monitoring of oropharyngeal pH and detection of pepsin in the middle ear fluid are suitable methods for detecting EER in children with OME. Patients with a positive Peptest and patients with a RYAN composite score above 200 have most severe EER and could be possibly the best candidates for antireflux treatment.
